# Effect of Various Acid Pretreatments on the Bonding Performance of Methyl Methacrylate-Based Resin Cement to Cobalt-Chromium Alloys

**DOI:** 10.7759/cureus.117055

**Published:** 2026-09-28

**Authors:** Kento Yamada, Koji Takahashi, So Koizumi, Katsura Ohashi, Tomotaro Nihei, Tetsutaro Yamaguchi

**Affiliations:** 1 Department of Orthodontics, School of Dentistry, Kanagawa Dental University, Yokosuka, JPN; 2 Department of Clinical Biomaterials, School of Dentistry, Kanagawa Dental University, Yokosuka, JPN

**Keywords:** acid treatment, additive manufacturing, bond strength, cobalt-chromium alloy, resin cement

## Abstract

Objective

Three-dimensional (3D)-printed cobalt-chromium (Co-Cr) alloys are increasingly used in dentistry, but durable bonding to resin cements remains challenging. The purpose of this study was to evaluate the bonding performance of a methyl methacrylate (MMA)-based resin cement to additively manufactured and cast Co-Cr alloys after different acid surface treatments.

Materials and methods

Co-Cr alloy specimens were fabricated by additive manufacturing or conventional casting, polished, and airborne-particle abraded. For each fabrication method, the specimens were assigned to five surface treatment groups: control (no acid treatment), S-Clean S-24 (SC), sulfuric acid (H₂SO₄), hydrochloric acid (HCl), and a hydrofluoric acid-containing porcelain etchant (BP). For shear bond strength (SBS) evaluation, each surface treatment group was further divided into four storage period subgroups: immediate, one day, seven days, and 28 days. An MMA-based resin cement was used for bonding. SBS was measured immediately after bonding and after one, seven, and 28 days of storage in deionized water at 37°C. Wettability was assessed by contact-angle measurements using distilled water and diiodomethane. Surface elemental composition and oxide-film thickness were evaluated by X-ray photoelectron spectroscopy (XPS). After SBS testing, failure modes were assessed under a stereomicroscope.

Statistical analyses were performed using R Version 4.5.1 (R Foundation for Statistical Computing, Vienna, Austria). SBS data were analyzed using three-way analysis of variance (ANOVA), and contact-angle data were analyzed using two-way ANOVA, with Tukey's honestly significant difference used for post hoc comparisons. Statistical significance was set at p<0.05.

Results

Surface treatment significantly affected SBS. The H₂SO₄- and HCl-treated groups generally exhibited higher SBS than the other treatment groups during the first seven days of water storage; however, SBS decreased in all groups by 28 days, with no significant differences among surface treatments at that time point. XPS revealed oxide-film thicknesses of approximately 1.7-5.1 nm, with specimens analyzed 24 hours after acid treatment tending to have thicker oxide films than those analyzed immediately after treatment. Interfacial failure predominated in both fabrication groups, whereas mixed failure was observed in only a small number of specimens, and no cohesive failure within the resin cement was observed.

Conclusions

H₂SO₄ and HCl surface treatments improved the initial bonding performance of MMA-based resin cement to cast and additively manufactured Co-Cr alloys. However, SBS decreased during water storage, and no significant differences among surface treatments remained after 28 days. These findings suggest that acid treatment can improve initial bonding but may be insufficient to ensure long-term bond durability.

## Introduction

In recent years, numerous studies have investigated bonding between resin-based materials and dental metals [1], and various surface treatment methods have been proposed to improve bond durability [2,3]. Resin bonding to metallic substrates is generally considered to depend on both micromechanical retention and chemical bonding, and it is well established that metal surface roughness and oxide layer characteristics substantially influence bonding performance [4-6]. In particular, cobalt-chromium (Co-Cr) alloys form a chromium oxide (Cr₂O₃)-based passive surface layer, which has been reported to participate in chemical interactions with functional resin monomers [7]. In addition to surface treatment, the composition of the resin cement is also an important factor in establishing adhesion to Co-Cr alloys. The methyl methacrylate (MMA)-based resin cement used in the present study contains 4-methacryloxyethyl trimellitate anhydride (4-META) as a functional monomer, which may contribute to bonding to Co-Cr alloy surfaces through interactions with the surface oxide layer [1]. Furthermore, surface treatments such as airborne-particle abrasion and acid etching alter the surface microstructure of metals, thereby enhancing micromechanical interlocking with resin cements [8].

In orthodontics, the increasing adoption of digital workflows has increased the use of computer-aided design/computer-aided manufacturing (CAD/CAM) and additive manufacturing technologies for the fabrication of metallic orthodontic appliances. These technologies enable the fabrication of appliances customized to individual dental arch morphology; however, appliance debonding remains an important clinical concern [9]. Co-Cr alloys can be used to fabricate fixed orthodontic appliances, such as lingual arches, Nance-holding arches, rapid palatal expanders, and maxillary protraction appliances [9]. Therefore, improving the bonding reliability between Co-Cr alloy orthodontic appliances and resin-based materials through appropriate surface treatment may enhance the predictability of orthodontic treatment.

Previous studies have suggested that acid treatment of metallic surfaces can modify surface characteristics and enhance mechanical retention, thereby influencing bond strength [10,11]. In particular, treatment with strong acids is considered to enhance mechanical retention at the bonding interface by modifying the surface oxide layer and creating microscopic surface irregularities [8,12].

Additively manufactured Co-Cr alloys have been increasingly used for the fabrication of fixed orthodontic appliances [9]. These alloys have been reported to possess microstructural and crystallographic characteristics that differ from those of conventionally cast alloys [13]. During additive manufacturing, repeated rapid melting and solidification induced by laser irradiation produce unique microstructural and surface characteristics that are distinct from those of cast alloys [14-16]. These differences may influence oxide layer formation and surface reactivity, thereby affecting the bonding behavior of resin cements to the alloy surface. However, most previous studies on metal-resin bonding have focused on conventionally cast alloys, and the effects of acid surface treatment on the bonding performance of resin cements to additively manufactured Co-Cr alloys remain insufficiently investigated. In particular, the influence of different acid treatments on the bond strength of additively manufactured Co-Cr alloys remains unclear, and further evidence is required to support their clinical application in orthodontic appliances [1].

Therefore, the purpose of the present study was to evaluate the bonding performance of an MMA-based resin cement to additively manufactured and cast Co-Cr alloys subjected to various acid surface treatments.

## Materials and methods

Materials

Additively manufactured Co-Cr alloy specimens were fabricated using the powder bed fusion laser beam technique from a Co-Cr alloy powder (SP2; EOS, Kreiling, Germany). Cylindrical specimens (20 mm in diameter and 1 mm thick) were designed using CAD software and exported as STL files. The specimens were produced using a metal 3D printer (EOSINT M 270 Dental; EOS, Kreiling, Germany). After fabrication, the specimens were detached from the build plate, and the support structures were removed. Cast Co-Cr alloy specimens were prepared from disk-shaped wax patterns (20 mm in diameter and 1 mm thick), which were invested according to the manufacturer's instructions and cast using a dental Co-Cr alloy (Biogil f; Dentsply Sirona, Tokyo, Japan). The following surface treatment agents were used: S-Clean S-24 (SC; Sasaki Chemical Co., Kyoto, Japan), hydrochloric acid (HCl; FUJIFILM Wako Pure Chemical Co., Osaka, Japan), sulfuric acid (H₂SO₄; FUJIFILM Wako Pure Chemical Co., Osaka, Japan), and BISCO porcelain etchant (BP; BISCO, Inc., Schaumburg, Illinois, United States) (Table 1). An MMA-based resin cement (Super-Bond; Sun Medical Co., Shiga, Japan) was used as the luting material (Table 2). The overall experimental design is summarized in Figure 1.

**Table 1:** Surface treatment agents

Surface treatment agent	Form	Composition	Abbreviation	Manufacturer	Lot number
S-Clean S-24	Liquid	Ammonium cerium(IV) nitrate, perchloric acid, and other ingredients	SC	Sasaki Chemical Co.	14012049
Hydrochloric acid	Liquid	Water, hydrogen chloride	HCl	FUJIFILM Wako Pure Chemical Co.	SEH4232
Sulfuric acid	Liquid	Sulfuric acid	H₂SO₄	FUJIFILM Wako Pure Chemical Co.	ELH4340
Porcelain etchant	Gel	Hydrofluoric acid, polyacrylamide, methylpropanesulfonic acid, water, and other ingredients	BP	BISCO, Inc.	2500001884

**Table 2:** Components of the MMA-based resin cement MMA: methyl methacrylate; 4-META: 4-methacryloxyethyl trimellitate anhydride; PMMA: polymethyl methacrylate

Component	Form	Composition	Abbreviation	Lot number
Quick monomer liquid	Liquid	MMA, 4-META, etc.	QM	KE12
Catalyst V	Liquid	Tri-n-butyl boron partial oxide, etc.	CV	GV1209
Polymer powder	Powder	PMMA, contains sodium fluoride	-	FKL1

**Figure 1:**
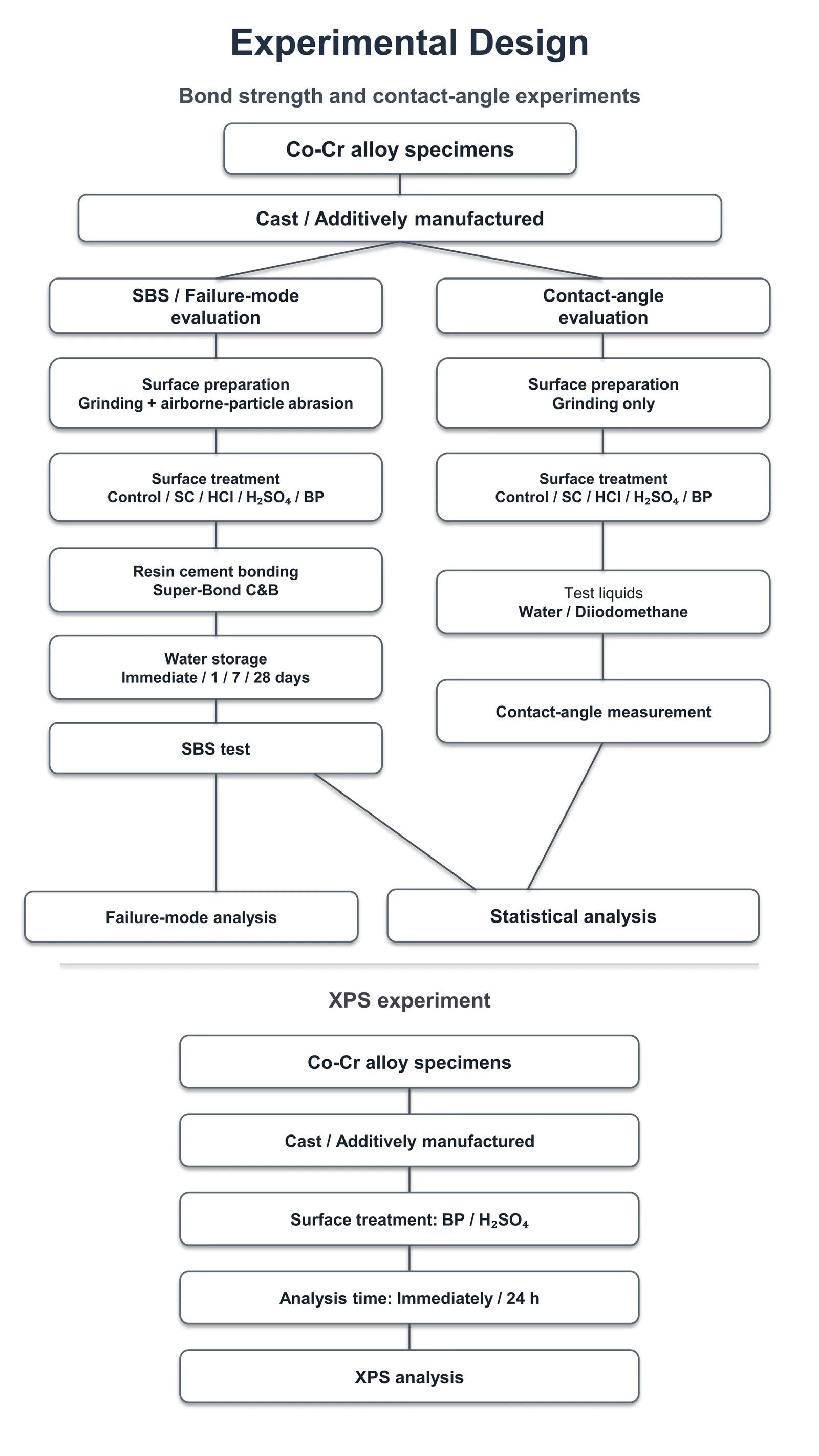
Experimental design of the study The flowchart summarizes the experimental procedures for SBS, failure mode, contact angle, and XPS analyses. SC: S-Clean S-24; HCl: hydrochloric acid; H₂SO₄: sulfuric acid; BP: BISCO porcelain etchant; SBS: shear bond strength; XPS: X-ray photoelectron spectroscopy; Co-Cr: cobalt-chromium

Experimental procedures

Bonding of Resin Cement to Co-Cr Alloys

Disk-shaped Co-Cr alloy specimens were embedded in autopolymerizing resin, leaving the bonding surface exposed by 1 mm. The bonding surfaces were sequentially polished with waterproof silicon carbide abrasive papers through #600 grit and then ultrasonically cleaned for 30 minutes. The specimens were then airborne-particle abraded with 250-μm alumina particles at a pressure of 0.2 MPa from a distance of 10 mm for 10 seconds. After airborne-particle abrasion, the specimens were ultrasonically cleaned again for 30 minutes and dried.

Grouping of the Specimens

For each fabrication method (cast and additively manufactured), the specimens were allocated to five surface treatment groups: control, SC, H₂SO₄, HCl, and BP. For shear bond strength (SBS) evaluation, each surface treatment group was further divided according to storage period into immediate, one-day, seven-day, and 28-day subgroups (n=12 per subgroup).

Surface Treatment and Bonding Procedures

The acid treatment groups consisted of SC, H₂SO₄, HCl, and BP. SC, H₂SO₄, and HCl were dispensed onto the metal surface using a pipette and then spread uniformly over the surface using a microbrush. BP was dispensed directly onto the metal surface using an applicator tip and then spread uniformly using a microbrush. Each acid treatment agent was left on the surface for 90 seconds, after which the specimens were thoroughly rinsed with running distilled water and air-dried.

To standardize the bonding area, a piece of mending tape (3M, St. Paul, Minnesota, United States) containing a circular hole 3 mm in diameter was applied to the bonding surface. A brass mold (3 mm in height, 5 mm in internal diameter, and 7 mm in external diameter) was then positioned over the specimen, and the resin cement was packed into the mold using the brush-dip technique. A static load of 1 kg was applied through a glass slide for 7 minutes. SBS was measured either immediately after bonding or after storage in deionized water at 37°C for one, seven, or 28 days using a universal testing machine (EZ-S; Shimadzu Corp., Kyoto, Japan) equipped with a metal knife-edge loading device at a crosshead speed of 1 mm/min. The loading device was positioned adjacent to the Co-Cr alloy-resin cement interface, and the load was applied perpendicular to the long axis of the resin cement cylinder until failure occurred.

After SBS testing, the debonded metal surfaces were examined under a stereomicroscope (SZ61; Olympus, Tokyo, Japan) at ×20 magnification to determine the failure mode. Failure modes were categorized as interfacial, mixed, or cohesive failure within the resin cement based on previously reported morphological criteria [4] (Table 3).

**Table 3:** Classification of failure modes Co-Cr: cobalt-chromium

Failure mode	Definition
Interfacial failure	Failure at the Co-Cr alloy-resin cement interface
Mixed failure	Failure involving both the Co-Cr alloy-resin cement interface and the resin cement
Cohesive failure within resin cement	Failure occurring within the resin cement

Surface Analysis of Co-Cr Alloys

Surface elemental composition and chemical bonding states were analyzed using X-ray photoelectron spectroscopy (XPS). Measurements were performed using a scanning XPS system (PHI 5000 VersaProbe III; ULVAC-PHI, Kanagawa, Japan). An Al Kα X-ray source (1486.6 eV) was used at a power of 25.1 W and with a beam diameter of 100 μm. Photoelectrons were collected at a take-off angle of 45° relative to the specimen surface. Charge neutralization was used to compensate for surface charging, and depth profiling was performed using Ar⁺ ion sputtering. After wide-scan spectra of the outermost surface were acquired, high-resolution spectra of the selected elements were obtained to evaluate changes in elemental composition and oxide layer thickness with depth. Both cast and additively manufactured Co-Cr alloy specimens were analyzed. Surface treatment was limited to the H₂SO₄ and BP groups, which were treated for 90 seconds, followed by rinsing with distilled water and drying. To investigate time-dependent changes in the surface oxide layer after acid treatment, two experimental conditions were evaluated: specimens analyzed immediately after acid treatment and specimens stored in air at room temperature for 24 hours before XPS analysis. Eight specimens were used for XPS analysis: four cast Co-Cr alloy specimens and four additively manufactured Co-Cr alloy specimens. For each fabrication method, two specimens were assigned to the H₂SO₄ treatment group and two to the BP treatment group. Each specimen was treated for 90 seconds, thoroughly rinsed with distilled water, and dried. To evaluate time-dependent changes in the surface condition after acid treatment, one specimen from each surface treatment group was analyzed by XPS immediately after treatment, whereas the remaining specimen was stored at room temperature in ambient air for 24 hours before analysis. One specimen (n=1) was analyzed for each combination of fabrication method, surface treatment, and analysis time point.

Contact-Angle Measurement

Surface wettability was evaluated by static contact-angle measurement using a contact angle analyzer (FAMAS; Kyowa Interface Science Co., Saitama, Japan). Distilled water and diiodomethane were used as probe liquids. A droplet of each liquid was placed on the specimen surface using a microsyringe, and the left and right contact angles were measured 10 seconds after droplet deposition. The mean of the two measurements was recorded as the contact angle at each measurement point. Ten specimens were evaluated for each group.

Statistical Analysis

Statistical analyses were performed using R (Version 4.5.1; R Foundation for Statistical Computing, Vienna, Austria). For SBS, three-way analysis of variance (ANOVA) was used to evaluate the effects of fabrication method, surface treatment, and storage period, as well as their interactions. Significant effects were followed by multiple comparisons using Tukey's honestly significant difference (HSD) test. For contact-angle measurements, data obtained using distilled water and diiodomethane were analyzed separately by two-way ANOVA, with fabrication method and surface treatment as the main factors. Significant effects were followed by post hoc multiple comparisons using Tukey's HSD test. For all analyses, p-values below 0.05 were considered statistically significant. Failure-mode distributions and XPS data were summarized descriptively and were not subjected to inferential statistical analysis.

## Results

SBS

The SBS results for each Co-Cr alloy are presented in Figure 2A-2B and Figure 3A-3B, as well as in Tables 4-7. Three-way ANOVA revealed significant main effects of fabrication method (F(1,440)=21.26; p<0.001; partial η²=0.05), surface treatment (F(4,440)=84.86; p<0.001; partial η²=0.44), and storage period (F(3,440)=427.47; p<0.001; partial η²=0.74). A significant surface treatment×storage period interaction was also observed (F(12,440)=15.44; p<0.001; partial η²=0.30). In contrast, the fabrication method×surface treatment (F(4,440)=0.53; p=0.717; partial η²<0.01), fabrication method×storage period (F(3,440)=0.70; p=0.553; partial η²<0.01), and three-way interaction (F(12,440)=0.84; p=0.605; partial η²=0.02) were not significant.

**Figure 2:**
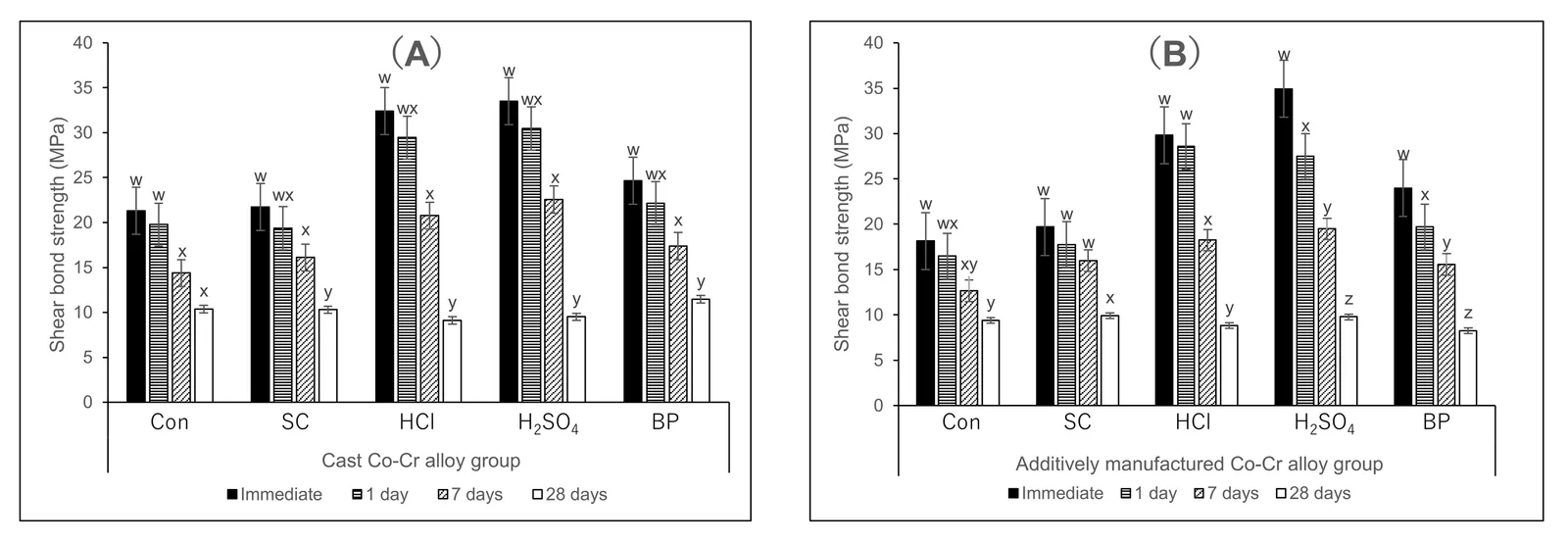
Shear bond strength of resin cement to cast and additively manufactured Co-Cr alloys across storage periods: (A) cast Co-Cr alloy group and (B) additively manufactured Co-Cr alloy group Different lowercase letters (w, x, y, and z) within each surface treatment group indicate statistically significant differences among storage periods (Tukey's honestly significant difference test; p<0.05). Data are presented as mean±SD; n=12 per group. Con: control; SC: S-Clean S-24; HCl: hydrochloric acid; H₂SO₄: sulfuric acid; BP: BISCO porcelain etchant; Co-Cr: cobalt-chromium

**Figure 3:**
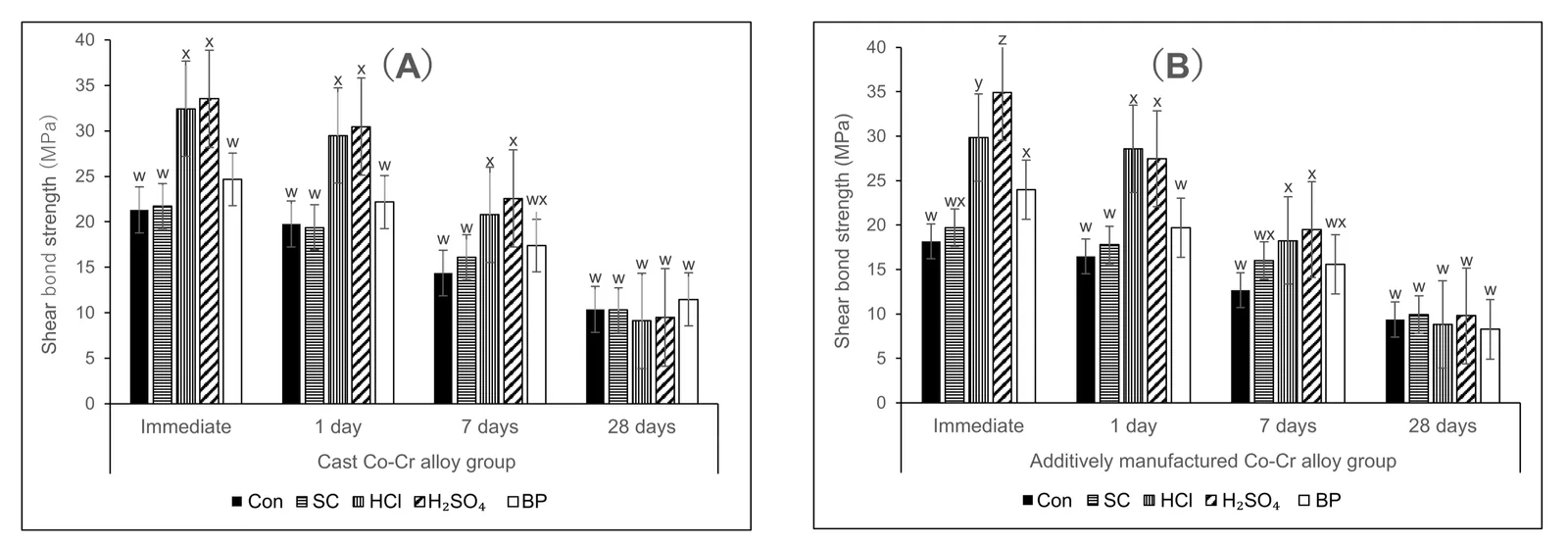
Shear bond strength of resin cement to cast and additively manufactured Co-Cr alloys across surface treatment groups: (A) cast Co-Cr alloy group and (B) additively manufactured Co-Cr alloy group Different lowercase letters (w, x, y, and z) within each storage period indicate statistically significant differences among surface treatment groups (Tukey's honestly significant difference test; p<0.05). Data are presented as mean±SD; n=12 per group. Con: control; SC: S-Clean S-24; HCl: hydrochloric acid; H₂SO₄: sulfuric acid; BP: BISCO porcelain etchant; Co-Cr: cobalt-chromium

**Table 4:** Shear bond strength according to fabrication method, surface treatment, and storage period Data are presented as mean±SD; n=12 per group. The 95% confidence intervals of the means were calculated using the t distribution. SD: standard deviation; SC: S-Clean S-24; HCl: hydrochloric acid; H₂SO₄: sulfuric acid; BP: BISCO porcelain etchant; Co-Cr: cobalt-chromium

Fabrication method	Surface treatment	Storage period	95% CI of the mean (MPa)	Mean±SD (MPa)
Cast Co-Cr alloy	Control	Immediate	17.44-25.19	21.31±6.10
		1 day	18.06-21.64	19.85±2.81
		7 days	12.02-16.71	14.36±3.69
		28 days	8.21-12.51	10.36±3.38
	SC	Immediate	19.68-23.77	21.73±3.22
		1 day	17.41-21.35	19.38±3.10
		7 days	13.48-18.71	16.09±4.12
		28 days	8.24-12.32	10.28±3.21
	H₂SO₄	Immediate	30.99-36.06	33.52±4.00
		1 day	27.62-33.33	30.48±4.49
		7 days	20.06-25.08	22.57±3.95
		28 days	7.46-11.52	9.49±3.20
	HCl	Immediate	29.91-34.95	32.43±3.97
		1 day	26.65-32.30	29.48±4.45
		7 days	18.23-23.30	20.77±4.00
		28 days	6.79-11.43	9.11±3.65
	BP	Immediate	23.02-26.31	24.66±2.59
		1 day	19.58-24.74	22.16±4.06
		7 days	14.83-19.94	17.38±4.02
		28 days	9.19-13.74	11.46±3.58
Additively manufactured Co-Cr alloy	Control	Immediate	15.80-20.51	18.16±3.71
		1 day	14.55-18.40	16.48±3.03
		7 days	9.72-15.63	12.67±4.65
		28 days	7.21-11.54	9.38±3.41
	SC	Immediate	18.79-20.64	19.71±1.45
		1 day	15.86-19.67	17.76±3.00
		7 days	13.35-18.64	15.99±4.17
		28 days	8.04-11.79	9.92±2.95
	H₂SO₄	Immediate	32.26-37.60	34.93±4.20
		1 day	25.10-29.87	27.49±3.76
		7 days	17.13-21.83	19.48±3.70
		28 days	7.93-11.64	9.78±2.92
	HCl	Immediate	27.53-32.14	29.84±3.63
		1 day	26.18-30.94	28.56±3.74
		7 days	15.81-20.70	18.25±3.85
		28 days	6.53-11.09	8.81±3.59
	BP	Immediate	21.57-26.38	23.98±3.78
		1 day	15.88-23.50	19.69±6.00
		7 days	12.53-18.62	15.57±4.79
		28 days	6.03-10.48	8.25±3.50

**Table 5:** Three-way ANOVA results for shear bond strength A three-way ANOVA was performed with fabrication method, surface treatment, and storage period as fixed factors, including all interactions. Statistical significance was set at p<0.05. The denominator degrees of freedom were 440 for all effects. df: degrees of freedom; partial η²: partial eta-squared; ANOVA: analysis of variance

Effect	df	F statistic	P-value	Partial η²
Fabrication method	1	21.26	<0.001	0.05
Surface treatment	4	84.86	<0.001	0.44
Storage period	3	427.47	<0.001	0.74
Fabrication method×surface treatment	4	0.53	0.717	<0.01
Fabrication method×storage period	3	0.70	0.553	<0.01
Surface treatment×storage period	12	15.44	<0.001	0.30
Fabrication method×surface treatment×storage period	12	0.84	0.605	0.02

**Table 6:** Pairwise comparisons of shear bond strength Mean differences were calculated as the first condition minus the second condition shown in each pairwise comparison. For fabrication method comparisons, mean differences were calculated as additively manufactured Co-Cr alloy minus cast Co-Cr alloy; negative values indicate lower values for the additively manufactured Co-Cr alloy, whereas positive values indicate higher values. P-values for surface treatment and storage period comparisons were adjusted using Tukey's method. For fabrication method comparisons, a single contrast between the additively manufactured and cast Co-Cr alloys was performed within each surface-treatment-by-storage-period combination. The degrees of freedom were 440 for all pairwise comparisons. Statistical significance was set at p<0.05. N/A indicates that the corresponding factor was not applicable because its levels are specified in the pairwise comparison. SC: S-Clean S-24; HCl: hydrochloric acid; H₂SO₄: sulfuric acid; BP: BISCO porcelain etchant; Co-Cr: cobalt-chromium; N/A: not applicable

Comparison type	Fabrication method	Storage period	Surface treatment	Pairwise comparison	Mean difference (MPa)	t statistic	P-value
Surface treatment comparison	Additively manufactured Co-Cr alloy	Immediate	N/A	Control – SC	-1.56	-1.00	0.855
				Control – H₂SO₄	-16.77	-10.75	<0.001
				Control – HCl	-11.68	-7.49	<0.001
				Control – BP	-5.82	-3.73	0.002
				SC – H₂SO₄	-15.21	-9.75	<0.001
				SC – HCl	-10.12	-6.49	<0.001
				SC – BP	-4.26	-2.73	0.051
				H₂SO₄ – HCl	5.09	3.26	0.010
				H₂SO₄ – BP	10.95	7.02	<0.001
				HCl – BP	5.86	3.75	0.002
	Cast Co-Cr alloy			Control – SC	-0.41	-0.26	0.999
				Control – H₂SO₄	-12.21	-7.83	<0.001
				Control – HCl	-11.11	-7.12	<0.001
				Control – BP	-3.35	-2.15	0.202
				SC – H₂SO₄	-11.80	-7.56	<0.001
				SC – HCl	-10.70	-6.86	<0.001
				SC – BP	-2.94	-1.88	0.328
				H₂SO₄ – HCl	1.10	0.70	0.955
				H₂SO₄ – BP	8.86	5.68	<0.001
				HCl – BP	7.76	4.98	<0.001
	Additively manufactured Co-Cr alloy	1 day		Control – SC	-1.29	-0.83	0.923
				Control – H₂SO₄	-11.01	-7.06	<0.001
				Control – HCl	-12.08	-7.74	<0.001
				Control – BP	-3.22	-2.06	0.239
				SC – H₂SO₄	-9.72	-6.23	<0.001
				SC – HCl	-10.79	-6.92	<0.001
				SC – BP	-1.93	-1.24	0.731
				H₂SO₄ – HCl	-1.07	-0.69	0.959
				H₂SO₄ – BP	7.79	5.00	<0.001
				HCl – BP	8.87	5.68	<0.001
	Cast Co-Cr alloy			Control – SC	0.47	0.30	0.998
				Control – H₂SO₄	-10.63	-6.81	<0.001
				Control – HCl	-9.63	-6.17	<0.001
				Control – BP	-2.31	-1.48	0.574
				SC – H₂SO₄	-11.10	-7.12	<0.001
				SC – HCl	-10.10	-6.47	<0.001
				SC – BP	-2.79	-1.79	0.382
				H₂SO₄ – HCl	1.00	0.64	0.968
				H₂SO₄ – BP	8.31	5.33	<0.001
				HCl – BP	7.31	4.69	<0.001
	Additively manufactured Co-Cr alloy	7 days		Control – SC	-3.32	-2.13	0.210
				Control – H₂SO₄	-6.81	-4.36	<0.001
				Control – HCl	-5.58	-3.58	0.004
				Control – BP	-2.90	-1.86	0.342
				SC – H₂SO₄	-3.49	-2.23	0.169
				SC – HCl	-2.26	-1.45	0.596
				SC – BP	0.42	0.27	0.999
				H₂SO₄ – HCl	1.23	0.79	0.935
				H₂SO₄ – BP	3.91	2.50	0.091
				HCl – BP	2.68	1.72	0.423
	Cast Co-Cr alloy			Control – SC	-1.73	-1.11	0.802
				Control – H₂SO₄	-8.21	-5.26	<0.001
				Control – HCl	-6.40	-4.10	<0.001
				Control – BP	-3.02	-1.93	0.301
				SC – H₂SO₄	-6.48	-4.15	<0.001
				SC – HCl	-4.67	-2.99	0.024
				SC – BP	-1.29	-0.83	0.923
				H₂SO₄ – HCl	1.81	1.16	0.775
				H₂SO₄ – BP	5.19	3.33	0.008
				HCl – BP	3.38	2.17	0.193
	Additively manufactured Co-Cr alloy	28 days		Control – SC	-0.54	-0.35	0.997
				Control – H₂SO₄	-0.41	-0.26	0.999
				Control – HCl	0.56	0.36	0.996
				Control – BP	1.12	0.72	0.952
				SC – H₂SO₄	0.13	0.09	1.000
				SC – HCl	1.10	0.71	0.955
				SC – BP	1.66	1.07	0.824
				H₂SO₄ – HCl	0.97	0.62	0.971
				H₂SO₄ – BP	1.53	0.98	0.865
				HCl – BP	0.56	0.36	0.997
	Cast Co-Cr alloy			Control – SC	0.09	0.06	1.000
				Control – H₂SO₄	0.87	0.56	0.981
				Control – HCl	1.26	0.81	0.929
				Control – BP	-1.10	-0.71	0.955
				SC – H₂SO₄	0.79	0.50	0.987
				SC – HCl	1.17	0.75	0.944
				SC – BP	-1.19	-0.76	0.941
				H₂SO₄ – HCl	0.39	0.25	0.999
				H₂SO₄ – BP	-1.97	-1.27	0.713
				HCl – BP	-2.36	-1.51	0.555
Storage period comparison	Additively manufactured Co-Cr alloy	N/A	Control	Immediate – 1 day	1.68	1.08	0.704
				Immediate – 7 days	5.48	3.51	0.003
				Immediate – 28 days	8.78	5.63	<0.001
				1 day – 7 days	3.80	2.44	0.072
				1 day – 28 days	7.10	4.55	<0.001
				7 days – 28 days	3.30	2.11	0.150
	Cast Co-Cr alloy			Immediate – 1 day	1.46	0.94	0.785
				Immediate – 7 days	6.95	4.45	<0.001
				Immediate – 28 days	10.95	7.02	<0.001
				1 day – 7 days	5.48	3.52	0.003
				1 day – 28 days	9.49	6.08	<0.001
				7 days – 28 days	4.00	2.57	0.052
	Additively manufactured Co-Cr alloy		SC	Immediate – 1 day	1.95	1.25	0.595
				Immediate – 7 days	3.72	2.39	0.081
				Immediate – 28 days	9.80	6.28	<0.001
				1 day – 7 days	1.77	1.13	0.668
				1 day – 28 days	7.85	5.03	<0.001
				7 days – 28 days	6.08	3.90	<0.001
	Cast Co-Cr alloy			Immediate – 1 day	2.35	1.51	0.435
				Immediate – 7 days	5.63	3.61	0.002
				Immediate – 28 days	11.45	7.34	<0.001
				1 day – 7 days	3.28	2.10	0.154
				1 day – 28 days	9.10	5.83	<0.001
				7 days – 28 days	5.82	3.73	0.001
	Additively manufactured Co-Cr alloy		H₂SO₄	Immediate – 1 day	7.44	4.77	<0.001
				Immediate – 7 days	15.45	9.90	<0.001
				Immediate – 28 days	25.14	16.12	<0.001
				1 day – 7 days	8.01	5.13	<0.001
				1 day – 28 days	17.70	11.35	<0.001
				7 days – 28 days	9.70	6.22	<0.001
	Cast Co-Cr alloy			Immediate – 1 day	3.05	1.95	0.207
				Immediate – 7 days	10.95	7.02	<0.001
				Immediate – 28 days	24.03	15.41	<0.001
				1 day – 7 days	7.90	5.07	<0.001
				1 day – 28 days	20.99	13.45	<0.001
				7 days – 28 days	13.08	8.39	<0.001
	Additively manufactured Co-Cr alloy		HCl	Immediate – 1 day	1.28	0.82	0.846
				Immediate – 7 days	11.58	7.42	<0.001
				Immediate – 28 days	21.02	13.48	<0.001
				1 day – 7 days	10.30	6.61	<0.001
				1 day – 28 days	19.75	12.66	<0.001
				7 days – 28 days	9.44	6.05	<0.001
	Cast Co-Cr alloy			Immediate – 1 day	2.95	1.89	0.234
				Immediate – 7 days	11.66	7.47	<0.001
				Immediate – 28 days	23.32	14.95	<0.001
				1 day – 7 days	8.71	5.58	<0.001
				1 day – 28 days	20.37	13.06	<0.001
				7 days – 28 days	11.66	7.47	<0.001
	Additively manufactured Co-Cr alloy		BP	Immediate – 1 day	4.29	2.75	0.032
				Immediate – 7 days	8.41	5.39	<0.001
				Immediate – 28 days	15.72	10.08	<0.001
				1 day – 7 days	4.12	2.64	0.042
				1 day – 28 days	11.44	7.33	<0.001
				7 days – 28 days	7.32	4.69	<0.001
	Cast Co-Cr alloy			Immediate – 1 day	2.50	1.60	0.378
				Immediate – 7 days	7.28	4.67	<0.001
				Immediate – 28 days	13.20	8.46	<0.001
				1 day – 7 days	4.78	3.07	0.012
				1 day – 28 days	10.70	6.86	<0.001
				7 days – 28 days	5.92	3.79	<0.001
Fabrication method comparison	N/A	Immediate	Control	Additively manufactured Co-Cr alloy – cast Co-Cr alloy	-3.16	-2.02	0.044
			SC	Additively manufactured Co-Cr alloy – cast Co-Cr alloy	-2.01	-1.29	0.198
			H₂SO₄	Additively manufactured Co-Cr alloy – cast Co-Cr alloy	1.40	0.90	0.369
			HCl	Additively manufactured Co-Cr alloy – cast Co-Cr alloy	-2.59	-1.66	0.098
			BP	Additively manufactured Co-Cr alloy – cast Co-Cr alloy	-0.69	-0.44	0.661
		1 day	Control	Additively manufactured Co-Cr alloy – cast Co-Cr alloy	-3.37	-2.16	0.031
			SC	Additively manufactured Co-Cr alloy – cast Co-Cr alloy	-1.61	-1.03	0.302
			H₂SO₄	Additively manufactured Co-Cr alloy – cast Co-Cr alloy	-2.99	-1.92	0.056
			HCl	Additively manufactured Co-Cr alloy – cast Co-Cr alloy	-0.92	-0.59	0.557
			BP	Additively manufactured Co-Cr alloy – cast Co-Cr alloy	-2.47	-1.58	0.114
		7 days	Control	Additively manufactured Co-Cr alloy – cast Co-Cr alloy	-1.69	-1.08	0.279
			SC	Additively manufactured Co-Cr alloy – cast Co-Cr alloy	-0.10	-0.06	0.949
			H₂SO₄	Additively manufactured Co-Cr alloy – cast Co-Cr alloy	-3.09	-1.98	0.048
			HCl	Additively manufactured Co-Cr alloy – cast Co-Cr alloy	-2.51	-1.61	0.108
			BP	Additively manufactured Co-Cr alloy – cast Co-Cr alloy	-1.81	-1.16	0.247
		28 days	Control	Additively manufactured Co-Cr alloy – cast Co-Cr alloy	-0.99	-0.63	0.527
			SC	Additively manufactured Co-Cr alloy – cast Co-Cr alloy	-0.36	-0.23	0.817
			H₂SO₄	Additively manufactured Co-Cr alloy – cast Co-Cr alloy	0.29	0.19	0.852
			HCl	Additively manufactured Co-Cr alloy – cast Co-Cr alloy	-0.29	-0.19	0.851
			BP	Additively manufactured Co-Cr alloy – cast Co-Cr alloy	-3.21	-2.06	0.040

**Table 7:** Relationship between surface treatment and SBS according to fabrication method Only experimental conditions showing statistically significant differences in SBS between the cast and additively manufactured Co-Cr alloy groups are presented. The t statistics and p-values were obtained from pairwise comparisons following three-way ANOVA (df=440). Co-Cr: cobalt-chromium; SBS,: shear bond strength; ANOVA: analysis of variance

Surface treatment	Storage period	Group with significantly higher SBS	t statistic	P-value
Control	Immediately	Cast Co-Cr alloy group had significantly higher SBS than the additively manufactured Co-Cr alloy group	-2.02	0.044
Control	1 day	Cast Co-Cr alloy group had significantly higher SBS than the additively manufactured Co-Cr alloy group	-2.16	0.031
Sulfuric acid	7 days	Cast Co-Cr alloy group had significantly higher SBS than the additively manufactured Co-Cr alloy group	-1.98	0.048
Porcelain etchant	28 days	Cast Co-Cr alloy group had significantly higher SBS than the additively manufactured Co-Cr alloy group	-2.06	0.040

In the cast Co-Cr alloy group, the HCl- and H₂SO₄-treated groups showed higher SBS immediately after bonding and after one day of water storage. At both time points, these groups had significantly higher SBS than the control, SC, and BP groups (all p<0.001), with no significant difference between the HCl- and H₂SO₄-treated groups (immediate: p=0.955; one day: p=0.968). After seven days of storage, the H₂SO₄-treated group showed significantly higher SBS than the control (p<0.001), SC (p<0.001), and BP (p=0.008) groups. The HCl-treated group also showed significantly higher SBS than the control (p<0.001) and SC (p=0.024) groups. After 28 days of storage, no significant differences were observed among the surface treatment groups (all p>0.05). Detailed pairwise comparisons, including t statistics and p-values, are presented in Table 6.

In the additively manufactured Co-Cr alloy group, the H₂SO₄-treated group showed significantly higher SBS immediately after bonding than the control, SC, and BP groups (all p<0.001) and the HCl-treated group (p=0.010). The HCl-treated group also showed significantly higher SBS than the control, SC, and BP groups (control and SC: p<0.001; BP: p=0.002). After one day of storage, the HCl- and H₂SO₄-treated groups showed significantly higher SBS than the control, SC, and BP groups (all p<0.001), with no significant difference between the HCl- and H₂SO₄-treated groups (p=0.959). After seven days, the H₂SO₄- and HCl-treated groups continued to show significantly higher SBS than the control group (p<0.001 and p=0.004, respectively), whereas no other pairwise comparisons were significant. After 28 days, no significant differences were observed among the surface treatment groups (all p>0.05).

Comparisons among storage periods showed treatment-dependent reductions in SBS. In both fabrication groups, the H₂SO₄- and HCl-treated groups showed significantly lower SBS at seven and 28 days than immediately after bonding or after one day of storage (all p<0.001), except for the immediate versus one-day comparison, which was not significant in the cast H₂SO₄-treated group (p=0.207) or in either HCl-treated group (additively manufactured: p=0.846; cast: p=0.234). In the control and SC groups, significant reductions were mainly observed after prolonged storage.

Comparison between fabrication methods revealed significantly lower SBS in the additively manufactured Co-Cr alloy than in the cast Co-Cr alloy under four conditions: the control group immediately after bonding (p=0.044) and after one day of storage (p=0.031), the H₂SO₄-treated group after seven days (p=0.048), and the BP-treated group after 28 days (p=0.040). No significant differences between fabrication methods were observed under the other experimental conditions (all p>0.05).

Contact angle

The contact-angle measurements following each acid treatment are presented in Figures 4-5 and Tables 8-11. For the water contact angle, two-way ANOVA revealed significant main effects of fabrication method (F(1,90)=14.68; p<0.001; partial η²=0.14) and surface treatment (F(4,90)=139.27; p<0.001; partial η²=0.86), as well as a significant interaction between fabrication method and surface treatment (F(4,90)=13.72; p<0.001; partial η²=0.38).

**Figure 4:**
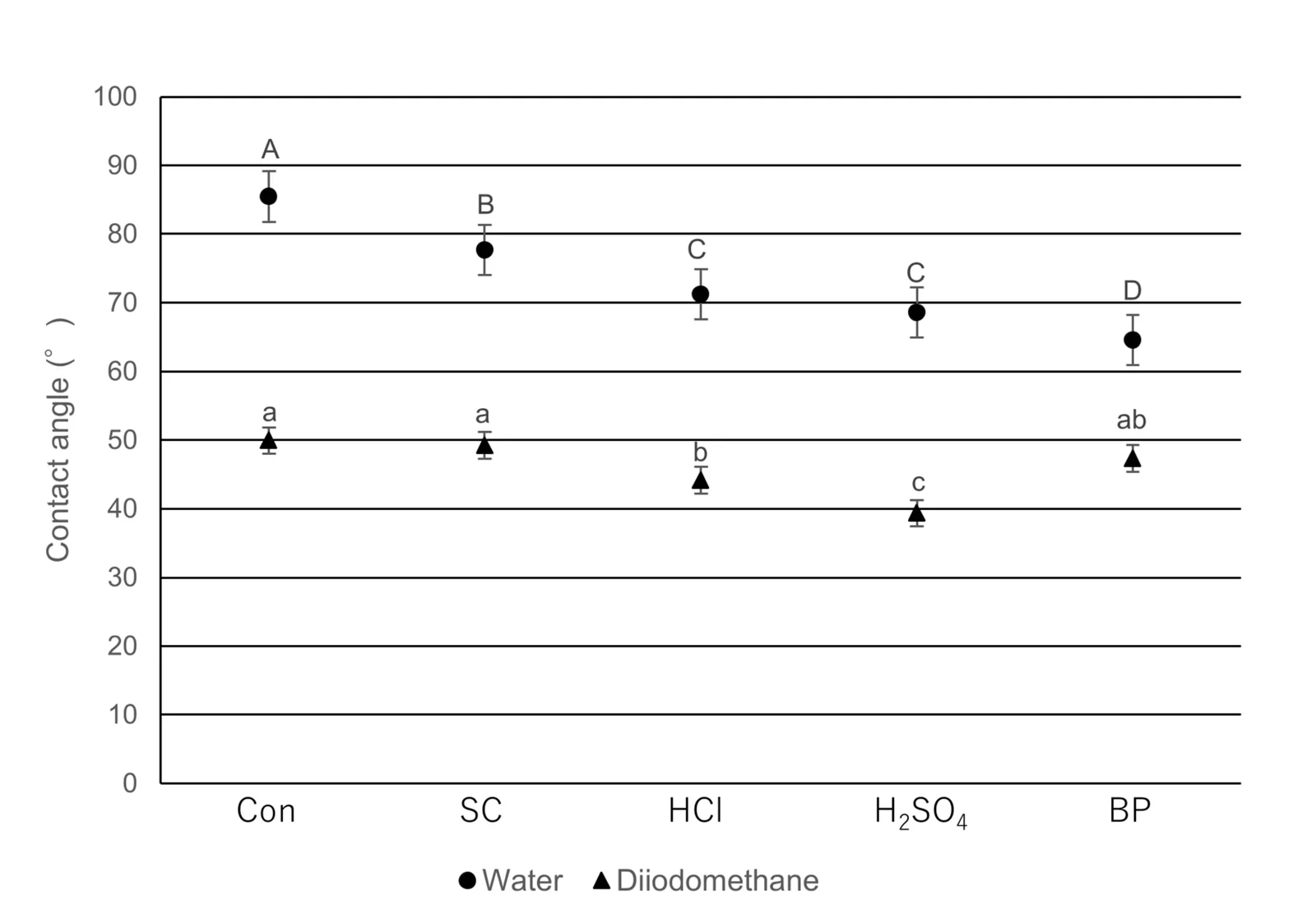
Contact angles of cast cobalt-chromium alloy surfaces measured using water and diiodomethane Different uppercase letters indicate statistically significant differences among surface treatment groups for water, whereas different lowercase letters indicate statistically significant differences among surface treatment groups for diiodomethane (Tukey's honestly significant difference test; p<0.05). Data are presented as mean±SD; n=10 per group. Con: control; SC: S-Clean S-24; HCl: hydrochloric acid; H₂SO₄: sulfuric acid; BP: BISCO porcelain etchant

**Figure 5:**
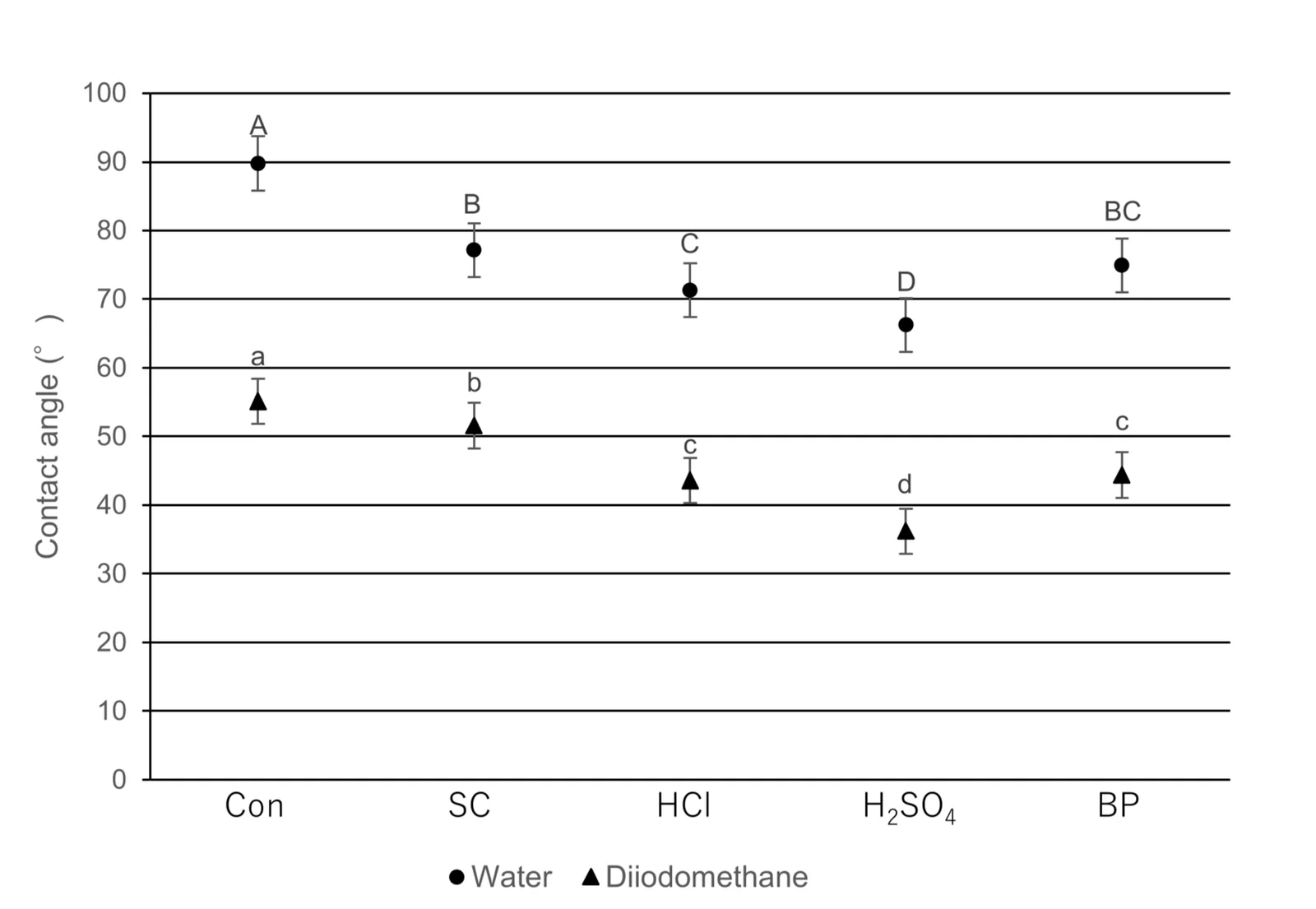
Contact angles of additively manufactured cobalt-chromium alloy surfaces measured using water and diiodomethane Different uppercase letters indicate statistically significant differences among surface treatment groups for water, whereas different lowercase letters indicate statistically significant differences among surface treatment groups for diiodomethane (Tukey's honestly significant difference test; p<0.05). Data are presented as mean±SD; n=10 per group. Con: control; SC: S-Clean S-24; HCl: hydrochloric acid; H₂SO₄: sulfuric acid; BP: BISCO porcelain etchant

**Table 8:** Contact angle according to fabrication method, surface treatment, and test liquid Data are presented as mean±SD; n=10 per group. The 95% confidence intervals of the means were calculated using the t distribution. SD: standard deviation; SC: S-Clean S-24; HCl: hydrochloric acid; H₂SO₄: sulfuric acid; BP: BISCO porcelain etchant; Co-Cr: cobalt-chromium

Liquid	Fabrication method	Surface treatment	95% CI of the mean (°)	Mean±SD (°)
Water	Cast Co-Cr alloy	Control	84.17-86.77	85.47±1.82
		SC	76.35-78.99	77.67±1.84
		HCl	67.90-74.62	71.26±4.69
		H₂SO₄	65.30-71.90	68.60±4.62
		BP	62.91-66.25	64.58±2.34
	Additively manufactured Co-Cr alloy	Control	88.36-91.22	89.79±2.00
		SC	75.96-78.30	77.13±1.63
		HCl	68.03-74.55	71.29±4.56
		H₂SO₄	64.46-68.00	66.23±2.47
		BP	73.24-76.62	74.93±2.36
Diiodomethane	Cast Co-Cr alloy	Control	48.18-51.68	49.93±2.44
		SC	48.04-50.46	49.25±1.69
		HCl	42.11-46.25	44.18±2.90
		H₂SO₄	37.27-41.41	39.34±2.89
		BP	46.24-48.42	47.33±1.53
	Additively manufactured Co-Cr alloy	Control	52.98-57.26	55.12±2.99
		SC	50.16-53.02	51.59±2.00
		HCl	41.47-45.71	43.59±2.97
		H₂SO₄	33.42-38.92	36.17±3.84
		BP	42.90-45.84	44.37±2.05

**Table 9:** Two-way ANOVA results for contact angle A two-way ANOVA was performed with fabrication method and surface treatment as fixed factors, including their interaction. The denominator degrees of freedom were 90 for all effects. Statistical significance was set at p<0.05. ANOVA: analysis of variance; df: degrees of freedom; partial η²: partial eta-squared

Liquid	Effect	df	F statistic	P-value	Partial η²
Water	Fabrication method	1	14.68	<0.001	0.14
	Surface treatment	4	139.27	<0.001	0.86
	Fabrication method×surface treatment	4	13.72	<0.001	0.38
Diiodomethane	Fabrication method	1	0.10	0.758	0.01
	Surface treatment	4	97.97	<0.001	0.81
	Fabrication method×surface treatment	4	9.36	<0.001	0.29

**Table 10:** Pairwise comparisons of water contact angle Mean differences were calculated as the first condition minus the second condition shown in each pairwise comparison. For fabrication method comparisons, mean differences were calculated as additively manufactured Co-Cr alloy minus cast Co-Cr alloy; negative values indicate lower values for the additively manufactured Co-Cr alloy, whereas positive values indicate higher values. P-values for surface treatment comparisons were adjusted using Tukey's method within each fabrication method. For fabrication method comparisons, a single contrast between the additively manufactured and cast Co-Cr alloys was performed within each surface treatment. The degrees of freedom were 90 for all pairwise comparisons. Statistical significance was set at p<0.05. SC: S-Clean S-24; HCl: hydrochloric acid; H₂SO₄: sulfuric acid; BP: BISCO porcelain etchant; Co-Cr: cobalt-chromium; N/A: not applicable

Fabrication method	Surface treatment	Pairwise comparison	Mean difference (°)	t statistic	P-value
Additively manufactured Co-Cr alloy	N/A	Control – SC	12.66	9.20	<0.001
		Control – HCl	18.50	13.44	<0.001
		Control – H₂SO₄	23.56	17.12	<0.001
		Control – BP	14.86	10.80	<0.001
		SC – HCl	5.84	4.24	<0.001
		SC – H₂SO₄	10.90	7.92	<0.001
		SC – BP	2.20	1.60	0.502
		HCl – H₂SO₄	5.06	3.68	0.004
		HCl – BP	-3.64	-2.65	0.071
		H₂SO₄ – BP	-8.70	-6.32	<0.001
Cast Co-Cr alloy		Control – SC	7.80	5.67	<0.001
		Control – HCl	14.21	10.33	<0.001
		Control – H₂SO₄	16.87	12.26	<0.001
		Control – BP	20.89	15.18	<0.001
		SC – HCl	6.41	4.66	<0.001
		SC – H₂SO₄	9.07	6.59	<0.001
		SC – BP	13.09	9.51	<0.001
		HCl – H₂SO₄	2.66	1.93	0.308
		HCl – BP	6.68	4.85	<0.001
		H₂SO₄ – BP	4.02	2.92	0.035
N/A	Control	Additively manufactured Co-Cr alloy – cast Co-Cr alloy	4.32	3.14	0.002
	SC		-0.54	-0.39	0.696
	HCl		0.03	0.02	0.983
	H₂SO₄		-2.37	-1.72	0.088
	BP		10.35	7.52	<0.001

**Table 11:** Pairwise comparisons of diiodomethane contact angle Mean differences were calculated as the first condition minus the second condition shown in each pairwise comparison. For fabrication method comparisons, mean differences were calculated as additively manufactured Co-Cr alloy minus cast Co-Cr alloy; negative values indicate lower values for the additively manufactured Co-Cr alloy, whereas positive values indicate higher values. P-values for surface treatment comparisons were adjusted using Tukey's method within each fabrication method. For fabrication method comparisons, a single contrast between the additively manufactured and cast Co-Cr alloys was performed within each surface treatment. The degrees of freedom were 90 for all pairwise comparisons. Statistical significance was set at p<0.05. SC: S-Clean S-24; HCl: hydrochloric acid; H₂SO₄: sulfuric acid; BP: BISCO porcelain etchant; Co-Cr: cobalt-chromium; N/A: not applicable

Fabrication method	Surface treatment	Pairwise comparison	Mean difference (°)	t statistic	P-value
Additively manufactured Co-Cr alloy	N/A	Control – SC	3.53	3.01	0.027
		Control – HCl	11.53	9.84	<0.001
		Control – H₂SO₄	18.95	16.17	<0.001
		Control – BP	10.75	9.17	<0.001
		SC – HCl	8.00	6.83	<0.001
		SC – H₂SO₄	15.42	13.16	<0.001
		SC – BP	7.22	6.16	<0.001
		HCl – H₂SO₄	7.42	6.33	<0.001
		HCl – BP	-0.78	-0.67	0.963
		H₂SO₄ – BP	-8.20	-7.00	<0.001
Cast Co-Cr alloy		Control – SC	0.68	0.58	0.978
		Control – HCl	5.75	4.91	<0.001
		Control – H₂SO₄	10.59	9.04	<0.001
		Control – BP	2.60	2.22	0.182
		SC – HCl	5.07	4.33	<0.001
		SC – H₂SO₄	9.91	8.46	<0.001
		SC – BP	1.92	1.64	0.477
		HCl – H₂SO₄	4.84	4.13	<0.001
		HCl – BP	-3.15	-2.69	0.064
		H₂SO₄ – BP	-7.99	-6.82	<0.001
N/A	Control	Additively manufactured Co-Cr alloy – cast Co-Cr alloy	5.19	4.43	<0.001
	SC		2.34	2.00	0.049
	HCl		-0.59	-0.50	0.616
	H₂SO₄		-3.17	-2.70	0.008
	BP		-2.96	-2.53	0.013

For the diiodomethane contact angle, two-way ANOVA revealed a significant main effect of surface treatment (F(4,90)=97.97; p<0.001; partial η²=0.81) and a significant interaction between fabrication method and surface treatment (F(4,90)=9.36; p<0.001; partial η²=0.29). In contrast, the main effect of fabrication method was not significant (F(1,90)=0.10; p=0.758; partial η²<0.01). Detailed pairwise comparisons, including t statistics and p-values, are presented in Tables 10 and 11.

In the cast Co-Cr alloy group, the water contact angle decreased in the following order: control > SC > HCl > H₂SO₄ > BP, with the BP-treated group showing the lowest value. The BP-treated group had a significantly lower water contact angle than the control, SC, and HCl groups (all p<0.001). The H₂SO₄-treated group also had a significantly lower water contact angle than the control and SC groups (both p<0.001) and the BP group (p=0.035). No significant difference was observed between the H₂SO₄- and HCl-treated groups (p=0.308).

In the additively manufactured Co-Cr alloy group, the water contact angle decreased in the following order: control > SC > BP > HCl > H₂SO₄, with the H₂SO₄-treated group showing the lowest value. The H₂SO₄-treated group had significantly lower water contact angles than the control, SC, and BP groups (all p<0.001). The HCl-treated group also had significantly lower water contact angles than the control and SC groups (both p<0.001), whereas no significant difference was observed between the HCl- and BP-treated groups (p=0.071).

Comparison between fabrication methods revealed significantly higher water contact angles in the additively manufactured Co-Cr alloy than in the cast Co-Cr alloy for the control (p=0.002) and BP (p<0.001) groups. No significant differences between fabrication methods were observed for SC (p=0.696), HCl (p=0.983), or H₂SO₄ (p=0.089).

For the diiodomethane contact angle, the H₂SO₄-treated group showed the lowest value in both fabrication groups. In the cast Co-Cr alloy group, the H₂SO₄-treated group had significantly lower contact angles than the control and SC groups (both p<0.001), and the HCl-treated group also had a significantly lower contact angle than the control group (p<0.001). In the additively manufactured Co-Cr alloy group, the H₂SO₄-treated group had significantly lower contact angles than the control, SC, and BP groups (all p<0.001). The HCl-treated group also had significantly lower contact angles than the control and SC groups (both p<0.001).

Comparison between fabrication methods revealed significantly higher diiodomethane contact angles in the additively manufactured Co-Cr alloy than in the cast Co-Cr alloy for the control (p<0.001) and SC (p=0.049) groups. In contrast, the cast Co-Cr alloy showed significantly higher contact angles than the additively manufactured Co-Cr alloy for the H₂SO₄ (p=0.008) and BP (p=0.013) groups. No significant difference between fabrication methods was observed for the HCl-treated group (p=0.616).

Failure modes

The failure modes after the SBS test are presented in Figures 6-7. Interfacial failure was the predominant failure mode in both the cast and additively manufactured Co-Cr alloy groups, whereas mixed failure was observed in only a small number of specimens. No cohesive failure within the resin cement was observed in either group.

**Figure 6:**
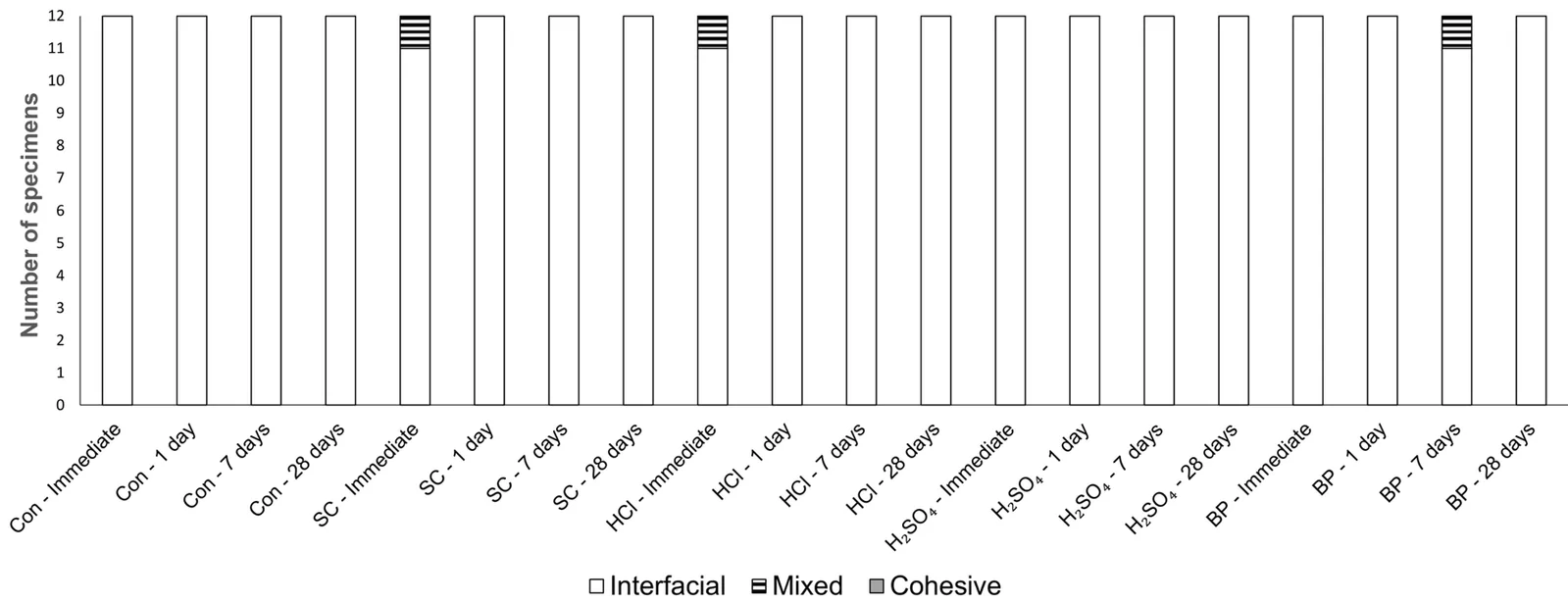
Failure mode distribution after shear bond strength testing of cast Co-Cr alloy Failure modes were classified as interfacial failure, mixed failure, or cohesive failure within the resin cement. Data represent the number of specimens in each failure mode; n=12 per experimental group. Con: control; SC: S-Clean S-24; HCl: hydrochloric acid; H₂SO₄: sulfuric acid; BP: BISCO porcelain etchant; Co-Cr: cobalt-chromium

**Figure 7:**
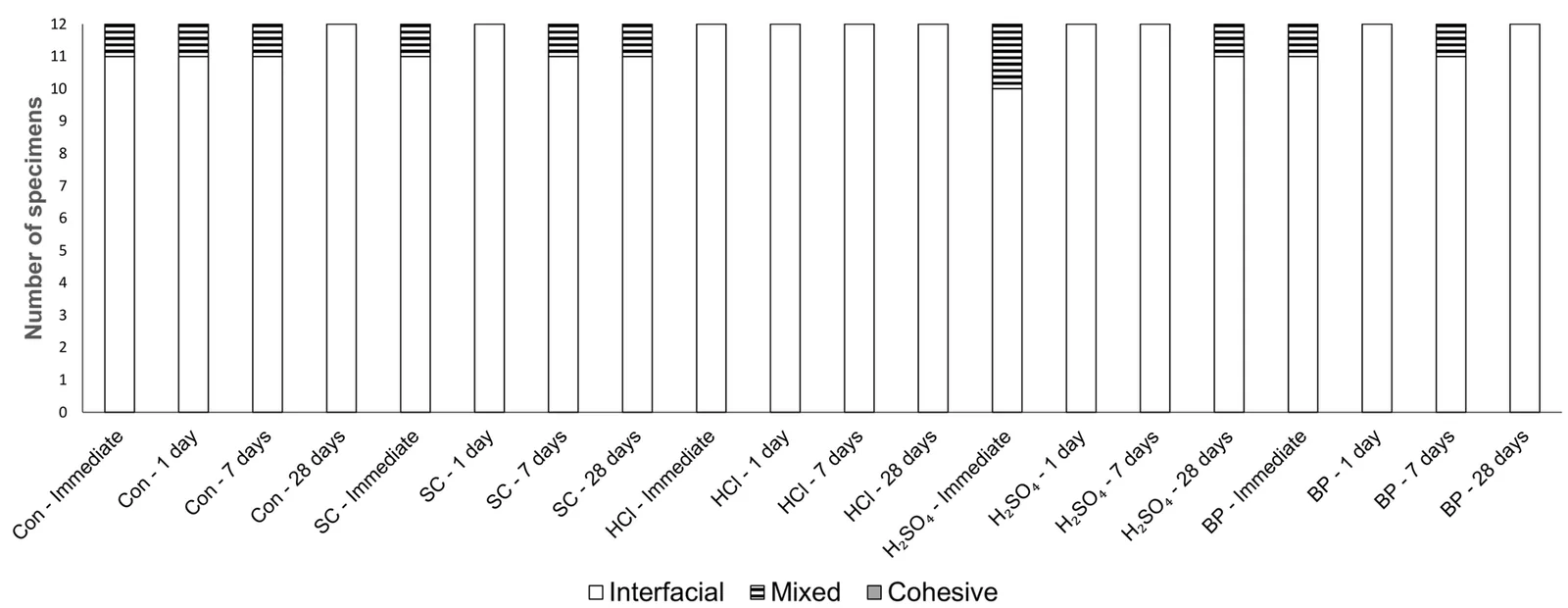
Failure mode distribution after shear bond strength testing of additively manufactured Co-Cr alloy Failure modes were classified as interfacial failure, mixed failure, or cohesive failure within the resin cement. Data represent the number of specimens in each failure mode; n=12 per experimental group. Con: control; SC: S-Clean S-24; HCl: hydrochloric acid; H₂SO₄: sulfuric acid; BP: BISCO porcelain etchant; Co-Cr: cobalt-chromium

Surface analysis of Co-Cr alloys

The oxide-film thicknesses calculated from the depth profile elemental analysis are shown in Figure 8A-8D and Figure 9A-9D.

**Figure 8:**
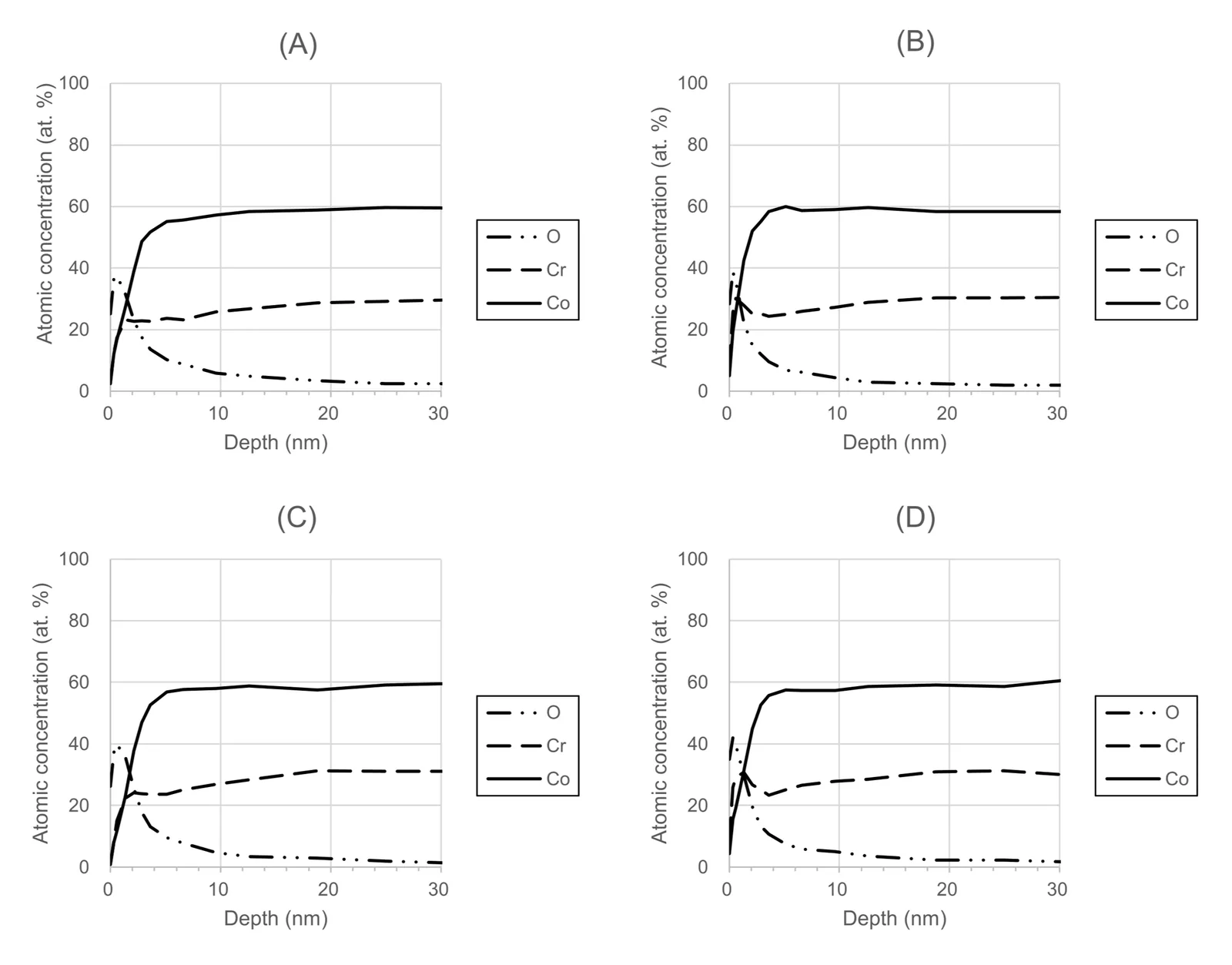
Depth profile analysis of elemental composition and oxide-film thickness in cast Co-Cr alloys using XPS (A) Cast Co-Cr alloy analyzed 24 hours after BP treatment; the estimated oxide-film thickness was 2.7 nm. (B) Cast Co-Cr alloy analyzed immediately after BP treatment; the estimated oxide-film thickness was 1.7 nm. (C) Cast Co-Cr alloy analyzed 24 hours after H₂SO₄ treatment; the estimated oxide-film thickness was 2.6 nm. (D) Cast Co-Cr alloy analyzed immediately after H₂SO₄ treatment; the estimated oxide-film thickness was 2 nm. The shaded region represents the estimated oxide-film region, extending from the specimen surface to the depth at which the O concentration decreased to the midpoint between zero and its maximum value. The numerical values shown in each panel indicate the oxide-film thickness, expressed as the SiO₂-equivalent thickness based on thermally oxidized silicon. One specimen was analyzed under each experimental condition (n=1 per condition; four specimens in total), and each panel shows the depth profile obtained from the corresponding specimen. XPS: X-ray photoelectron spectroscopy; Co-Cr: cobalt-chromium; BP: BISCO porcelain etchant; H₂SO₄: sulfuric acid; O: oxygen; Cr: chromium; Co: cobalt; SiO₂: silicon dioxide; at%: atomic percentage

**Figure 9:**
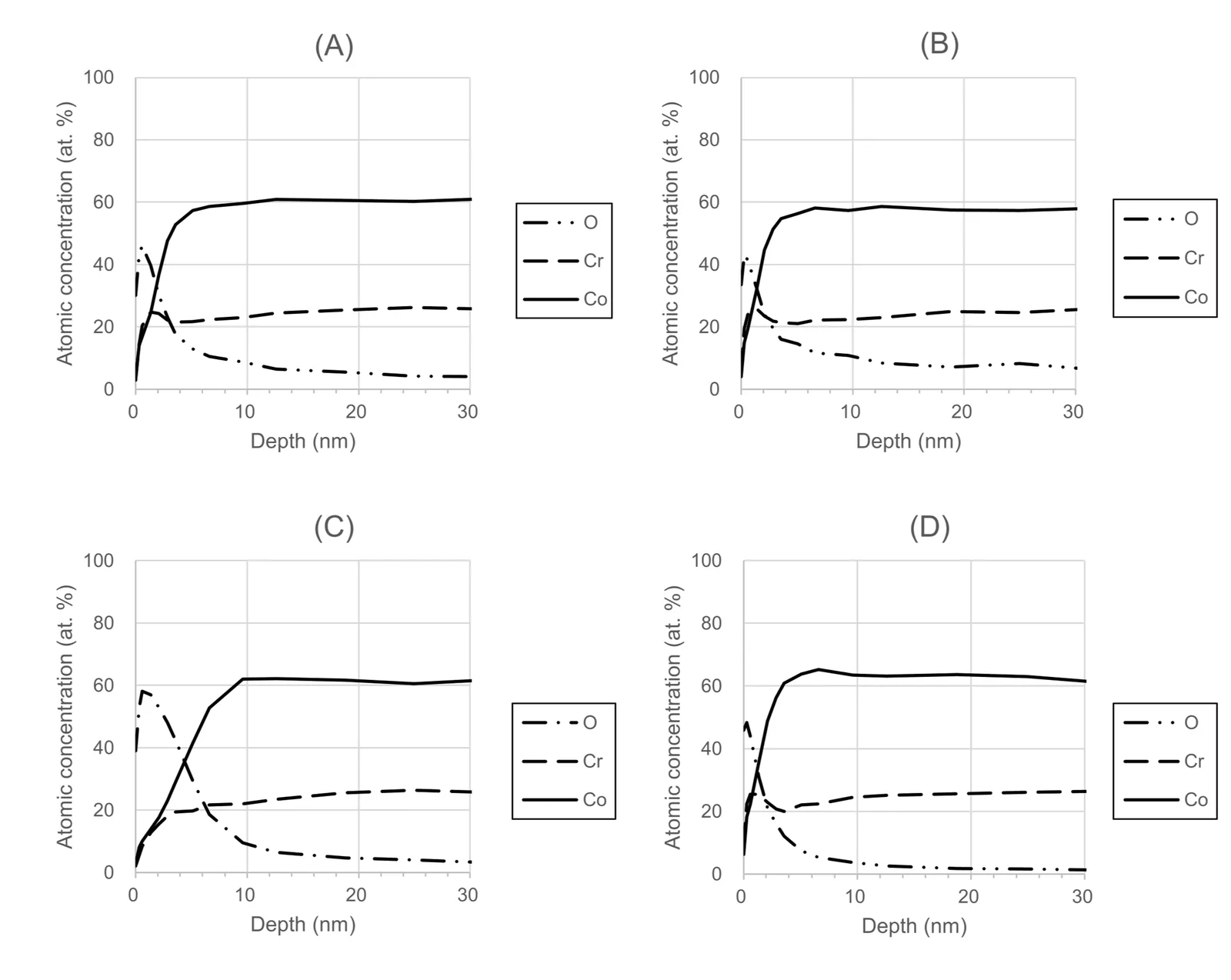
Depth profile analysis of elemental composition and oxide-film thickness in additively manufactured Co-Cr alloys using XPS (A) Additively manufactured Co-Cr alloy analyzed 24 hours after BP treatment; the estimated oxide-film thickness was 2.9 nm. (B) Additively manufactured Co-Cr alloy analyzed immediately after BP treatment; the estimated oxide-film thickness was 2.5 nm. (C) Additively manufactured Co-Cr alloy analyzed 24 hours after H₂SO₄ treatment; the estimated oxide-film thickness was 5.1 nm. (D) Additively manufactured Co-Cr alloy analyzed immediately after H₂SO₄ treatment; the estimated oxide-film thickness was 1.9 nm. The shaded region represents the estimated oxide-film region, extending from the specimen surface to the depth at which the O concentration decreased to the midpoint between zero and its maximum value. The numerical values shown in each panel indicate the oxide-film thickness, expressed as the SiO₂-equivalent thickness based on thermally oxidized silicon. One specimen was analyzed under each experimental condition (n=1 per condition; four specimens in total), and each panel shows the depth profile obtained from the corresponding specimen. XPS: X-ray photoelectron spectroscopy; Co-Cr: cobalt-chromium; BP: BISCO porcelain etchant; H₂SO₄: sulfuric acid; O: oxygen; Cr: chromium; Co: cobalt; SiO₂: silicon dioxide; at%: atomic percentage

In the cast Co-Cr alloy group, the oxide-film thickness in the BP-treated group was 2.7 nm 24 hours after acid treatment and 1.7 nm immediately after treatment. In the H₂SO₄-treated group, the oxide-film thickness was 2.6 nm after 24 hours and 2 nm immediately after treatment.

In the additively manufactured Co-Cr alloy group, the oxide-film thickness in the BP-treated group was 2.9 nm 24 hours after acid treatment and 2.5 nm immediately after treatment. In the H₂SO₄-treated group, the oxide-film thickness was 5.1 nm after 24 hours and 1.9 nm immediately after treatment.

Regardless of fabrication method, specimens analyzed immediately after acid treatment tended to have thinner oxide films than those analyzed 24 hours after treatment. Notably, the additively manufactured Co-Cr alloy treated with H₂SO₄ and analyzed 24 hours after treatment had the greatest oxide-film thickness (5.1 nm) among the specimens.

In summary, the principal findings were as follows: For SBS, surface treatment and storage period significantly affected SBS. H₂SO₄ and HCl treatments showed higher initial SBS values in several comparisons, although these differences diminished with storage. For contact angle, acid treatment reduced the contact angles of water and diiodomethane. For failure mode, interfacial failure was the predominant failure mode. For XPS analysis, the oxide films tended to be thicker 24 hours after acid treatment than immediately after treatment.

## Discussion

The present study investigated the effects of various acid treatments on the bonding performance of an MMA-based resin cement to cast and additively manufactured Co-Cr alloys. The bonding performance of 4-META-containing MMA-based resin cement to Co-Cr alloys has been reported previously [1]. In both the cast and additively manufactured groups, the H₂SO₄- and HCl-treated groups exhibited higher SBS from immediately after bonding through seven days of water storage, whereas SBS decreased in all groups after 28 days, with no significant differences remaining among the surface treatment groups (p>0.05). These findings are consistent with previous studies demonstrating that the bond strength between Co-Cr alloys and resin materials decreases with aging, including water storage and thermocycling, and that appropriate surface treatment can improve initial bond strength [1,17].

The higher initial SBS observed in the H₂SO₄- and HCl-treated groups may be attributed to acid-induced changes in the chemical state of the oxide layer and surface microtopography, which may have improved resin wettability and reduced interfacial defects. Although the substrate material differed from that used in the present study, previous investigations have shown that H₂SO₄/HCl etching following alumina sandblasting produces microscopic surface porosities, thereby enhancing resin bonding [11,18,19].

In contrast, the BP-treated groups generally exhibited lower mean SBS than the H₂SO₄- and HCl-treated groups from immediately after bonding through seven days of water storage. After 28 days, the cast BP-treated group showed a numerically higher SBS than the control group; however, no significant differences were observed among the surface treatment groups at this time point (p>0.05). Therefore, these findings do not demonstrate that BP treatment reduced the loss of bond strength over time. Because BP contains hydrofluoric acid, it may alter the oxide chemistry of the Co-Cr alloy surface [20]. Nevertheless, the present XPS analysis did not directly identify CrF₃ or establish an association between chromium fluoride formation and bond durability. Accordingly, the selective conversion of Cr₂O₃ to CrF₃ and the proposed stability of such a layer during water storage remain hypothetical and require direct chemical-state analysis and further investigation in dental Co-Cr alloys.

The contact-angle measurements also showed that BP treatment reduced the water contact angle, although the extent of this reduction differed between the cast and additively manufactured groups. The reduction in the diiodomethane contact angle appeared less pronounced with BP treatment than with H₂SO₄ or HCl treatment. These findings may reflect differences in the effects of BP treatment on the polar and dispersive components of surface free energy, which could influence the initial wettability of the MMA-based resin cement.

In the present study, acid treatment reduced both the water and diiodomethane contact angles. The decrease in the water contact angle suggests that the alloy surface became more hydrophilic, possibly because of an increased density of polar sites, such as hydroxyl (OH) groups, on the oxide layer. In contrast, because the diiodomethane contact angle is primarily influenced by the dispersive component of surface free energy, its reduction may reflect changes in dispersive surface interactions induced by acid treatment. Together, the reductions in water and diiodomethane contact angles may indicate an overall improvement in surface wettability. Improved wettability may facilitate the initial penetration of MMA-based resin cement and reduce interfacial void formation, consistent with the increased SBS observed from immediately after bonding through seven days of water storage [21]. Furthermore, the reductions in contact angle observed in both fabrication groups suggest that acid treatment may improve wettability, although the magnitude of this effect appears to depend on the fabrication method and surface treatment.

Differences in fabrication method may also influence bonding behavior. Additively manufactured Co-Cr alloys have been reported to exhibit manufacturing-related defects and oxide-film characteristics that differ from those of conventionally cast alloys, which may influence their bonding behavior. However, a previous study also reported comparable oxide-film thicknesses between cast and additively manufactured Co-Cr alloys [1].

In addition, differences in fabrication method have been reported to influence the surface roughness and oxide-film characteristics of Co-Cr alloys, which may consequently affect surface free energy and wettability following acid treatment [12,22]. A previous study demonstrated that different manufacturing techniques, including casting, milling, and selective laser sintering (SLS), influence surface characteristics and bonding performance [12].

Interfacial failure predominated in both fabrication groups, whereas mixed failure was observed in only a small number of specimens, and no cohesive failure within the resin cement was observed. This finding is consistent with a previous study reporting that interfacial failure is the predominant failure mode in resin bonding to Co-Cr alloys [1]. Notably, although acid treatment improved initial bond strength, the failure mode did not shift substantially toward cohesive failure within the resin cement. These findings suggest that the metal-resin interface remained the weakest region of the bonded assembly, which may have contributed to the reduction in SBS after 28 days of water storage.

The reduction in SBS observed after 28 days of water storage is likely attributable to multiple interacting factors. First, water absorption by MMA-based dental polymer matrices increases polymer-chain mobility, resulting in plasticization and deterioration of mechanical properties [23]. Such softening of the polymer matrix may reduce the cohesive strength of the resin cement and alter stress distribution at the adhesive interface, thereby contributing to bond degradation.

Second, the oxide layer modified by acid treatment may undergo gradual chemical changes during water storage through reactions with dissolved oxygen and water molecules, resulting in time-dependent alterations in surface hydroxyl concentration and chemical reactivity [24]. These changes in surface chemistry may weaken both chemical interactions and micromechanical interlocking at the metal-resin interface, thereby contributing to the long-term reduction in bond strength [1]. These proposed mechanisms are consistent with the present findings, in which the beneficial effects of acid treatment on initial bond strength gradually diminished during water storage, resulting in no significant differences among the treatment groups after 28 days.

These findings suggest that acid treatment alone may be insufficient to ensure long-term chemical stability of the bonding interface. Therefore, combining acid treatment with functional monomers, such as 10-methacryloyloxydecyl dihydrogen phosphate (MDP), which can establish covalent or coordination bonds with the metal surface, may help improve long-term bonding durability [25].

Previous studies have demonstrated that acidic primers and MDP-containing functional monomers can improve the durability of resin bonding to Co-Cr alloys [2,7].

To evaluate the chemical changes induced by acid treatment on the Co-Cr alloy surface, XPS was performed. The results confirmed the presence of an oxide film on the surface of all Co-Cr alloy specimens. The oxide-film thickness ranged from approximately 1.7 to 5.1 nm, indicating that a nanometer-scale oxide layer was present regardless of the treatment condition.

Specimens stored in air at room temperature for 24 hours after acid treatment tended to exhibit thicker oxide films than specimens analyzed immediately after treatment. This finding suggests that the surface oxide layer, which had been partially removed or chemically modified by acid treatment, underwent repassivation upon exposure to the atmosphere, resulting in the formation of a new oxide film [26]. Co-Cr alloys are well known for their excellent corrosion resistance, which is maintained by a passive film composed primarily of Cr₂O₃ [24,27]. Previous studies have demonstrated that this passive film is rapidly reformed after surface activation or mechanical or chemical damage. Consequently, the increase in oxide-film thickness observed after 24 hours of storage in the present study is consistent with these previous findings [28].

By contrast, no clear difference in oxide-film thickness was observed between the cast and additively manufactured Co-Cr alloys. Although additively manufactured Co-Cr alloys have been reported to possess a finer microstructure produced by rapid solidification, under the acid treatment conditions used in the present study, this microstructural difference did not result in a measurable difference in oxide-film thickness [29]. These findings suggest that the differences in bond strength observed between the cast and additively manufactured alloys cannot be explained by oxide-film thickness alone and may be influenced by multiple factors, including surface roughness, the chemical composition of the oxide layer, and chemical interactions between the oxide surface and resin monomers [1,3].

Furthermore, both the BP- and H₂SO₄-treated groups exhibited oxide films only a few nanometers thick, with no substantial difference between the two treatments. Nevertheless, significant differences in SBS were observed. These findings suggest that oxide-film thickness alone may not account for the differences in bonding performance and that other surface characteristics, including oxide-film composition, surface functional groups, and surface wettability, may also contribute to resin bonding [21].

The standardized in vitro design of this study enabled controlled comparison of acid pretreatments between cast and additively manufactured Co-Cr alloys. Nevertheless, direct extrapolation to clinically shaped orthodontic appliances should be made cautiously. The 28-day water storage period provided information on early bond durability, whereas longer aging, thermocycling, and cyclic loading would further clarify long-term performance. Because bond strength was evaluated only using an SBS test, the results may have been influenced by the test configuration. Future studies incorporating tensile or microtensile bond strength testing are warranted. Overall, the findings provide a basis for considering controlled extraoral HCl or H₂SO₄ pretreatment to improve the initial bonding of MMA-based resin cement to Co-Cr orthodontic components and support further evaluation under clinically relevant conditions.

## Conclusions

The present study evaluated the effects of various acid treatments on the bonding performance of an MMA-based resin cement to cast and additively manufactured Co-Cr alloys. H₂SO₄ and HCl treatments generally exhibited higher SBS from immediately after bonding through seven days of water storage, and the accompanying reductions in contact angle suggest that improved wettability may have contributed to the enhanced initial bond strength. After 28 days of water storage, SBS decreased in all groups, with no significant differences remaining among the surface treatment groups. Interfacial failure predominated in both fabrication groups, suggesting that the Co-Cr alloy-resin cement interface remained the weakest region of the bonded assembly and that acid treatment alone may be insufficient to ensure long-term stability of the bonding interface.

XPS analysis showed that, in both cast and additively manufactured Co-Cr alloys, specimens stored in air at room temperature for 24 hours after acid treatment tended to have thicker oxide films than those analyzed immediately after treatment. Overall, the pattern of bonding behavior was broadly similar between the cast and additively manufactured groups, suggesting that the effects of acid surface treatment were observed across both fabrication methods. Further studies are required to investigate surface treatment strategies, including their combined use with functional monomer-based primers, to improve the long-term durability of bonding between Co-Cr alloys and resin materials.

## References

[1] Takahashi K, Koizumi S, Ohashi K, Nihei T, Yamaguchi T (2024). Comparison of bond strength between two resin cement types and additive manufacturing or cast cobalt-chromium alloys. Cureus.

[2] Sadighpour L, Mostafavi AS, Pirmoradian M, Alipuryalda F (2021). Comparison of bond strength in the different class of resin cements to cast and CAD/CAM Co-Cr alloys. Int J Dent.

[3] Kern M, Thompson VP (1995). Durability of resin bonds to a cobalt-chromium alloy. J Dent.

[4] Shafiei F, Behroozibakhsh M, Abbasian A, Shahnavazi S (2018). Bond strength of self-adhesive resin cement to base metal alloys having different surface treatments. Dent Res J (Isfahan).

[5] Taira Y, Matsumura H, Yoshida K, Tanaka T, Atsuta M (1998). Influence of surface oxidation of titanium on adhesion. J Dent.

[6] Ohno H, Araki Y, Endo K, Kawashima I (1989). The adhesion mechanism of dental adhesive resin to the alloy: adhesive ability of dental adhesive resin to the cleaned metal surface obtained by hydrogen gas reduction method. Dent Mater J.

[7] Matsumura H, Tanaka T, Taira Y, Atsuta M (1996). Bonding of a cobalt-chromium alloy with acidic primers and tri-n-butylborane-initiated luting agents. J Prosthet Dent.

[8] Taira Y, Egoshi T, Kamada K, Sawase T (2014). Surface modification with alumina blasting and H2SO4-HCl etching for bonding two resin-composite veneers to titanium. Eur J Oral Sci.

[9] Takahashi K, Koizumi S, Shimura M, Seimiya K, Nihei T, Yamaguchi T (2026). Clinical evaluation of detachment and non-detachment of fixed orthodontic appliances fabricated using CAD/CAM technology. Clin Investig Orthod.

[10] Isidor F, Hassna NM, Josephsen K, Kaaber S (1991). Tensile bond strength of resin-bonded non-precious alloys with chemically and mechanically roughened surfaces. Dent Mater.

[11] Egoshi T, Taira Y, Soeno K, Sawase T (2013). Effects of sandblasting, H2SO4/HCl etching, and phosphate primer application on bond strength of veneering resin composite to commercially pure titanium grade 4. Dent Mater J.

[12] Ünalan Değirmenci B, Ersoy NM (2021). The effects of current production techniques on the surface roughness, oxide layer thickness and porcelain bond strength of cobalt-chromium and titanium substructures. Int Dent Res.

[13] Koutsoukis T, Zinelis S, Eliades G, Al-Wazzan K, Rifaiy MA, Al Jabbari YS (2015). Selective laser melting technique of Co-Cr dental alloys: a review of structure and properties and comparative analysis with other available techniques. J Prosthodont.

[14] Li J, Chen C, Liao J, Liu L, Ye X, Lin S, Ye J (2017). Bond strengths of porcelain to cobalt-chromium alloys made by casting, milling, and selective laser melting. J Prosthet Dent.

[15] Xiang N, Xin XZ, Chen J, Wei B (2012). Metal-ceramic bond strength of Co-Cr alloy fabricated by selective laser melting. J Dent.

[16] Xin XZ, Chen J, Xiang N, Wei B (2013). Surface properties and corrosion behavior of Co-Cr alloy fabricated with selective laser melting technique. Cell Biochem Biophys.

[17] Yoshida K, Taira Y, Sawase T, Atsuta M (1997). Effects of adhesive primers on bond strength of self-curing resin to cobalt-chromium alloy. J Prosthet Dent.

[18] Ferrari M, Cagidiaco MC, Borracchini A, Bertelli E (1989). Evaluation of a chemical etching solution for nickel-chromium-beryllium and chromium-cobalt alloys. J Prosthet Dent.

[19] Jokela-Hietamäki M, Rantanen T (1987). Resin bond to electrolytically etched cobalt-chromium alloys. Scand J Dent Res.

[20] Yang X, Xiang N, Wei B (2014). Effect of fluoride content on ion release from cast and selective laser melting-processed Co-Cr-Mo alloys. J Prosthet Dent.

[21] Marshall SJ, Bayne SC, Baier R, Tomsia AP, Marshall GW (2010). A review of adhesion science. Dent Mater.

[22] Yung KC, Xiao TY, Choy HS, Wang WJ, Cai ZX (2018). Laser polishing of additive manufactured CoCr alloy components with complex surface geometry. J Mater Process Technol.

[23] Rodrigues SA Jr, Scherrer SS, Ferracane JL, Della Bona A (2008). Microstructural characterization and fracture behavior of a microhybrid and a nanofill composite. Dent Mater.

[24] Hanawa T (2004). Metal ion release from metal implants. Mater Sci Eng C.

[25] Yanagida H, Tanoue N, Ide T, Matsumura H (2009). Evaluation of two dual-functional primers and a tribochemical surface modification system applied to the bonding of an indirect composite resin to metals. Odontology.

[26] Tsutsumi Y, Doi H, Nomura N, Ashida M, Chen P, Kawasaki A, Hanawa T (2016). Surface composition and corrosion resistance of Co-Cr alloys containing high chromium. Mater Trans.

[27] Qiao G, Su J, He M (2012). Effect of (-)-epigallocatechin gallate on electrochemical behavior and surface film composition of Co-Cr alloy used in dental restorations. Dent Mater J.

[28] Thornley B, Beadling R, Bryant M, Neville A (2020). Investigation of the repassivation process of CoCrMo in simulated biological fluids. Corrosion.

[29] Li KC, Prior DJ, Waddell JN, Swain MV (2015). Comparison of the microstructure and phase stability of as-cast, CAD/CAM and powder metallurgy manufactured Co-Cr dental alloys. Dent Mater.

